# The Effects of Temperature Curing on the Strength Development, Transport Properties, and Freeze-Thaw Resistance of Blast Furnace Slag Cement Mortars Modified with Nanosilica

**DOI:** 10.3390/ma13245800

**Published:** 2020-12-18

**Authors:** Karol Federowicz, Vitoria Alves Figueiredo, Hussein Al-kroom, Hamdy A. Abdel-Gawwad, Mohamed Abd Elrahman, Pawel Sikora

**Affiliations:** 1 Faculty of Civil and Environmental Engineering, West Pomeranian University of Technology Szczecin, 70-311 Szczecin, Poland; karol.federowicz@zut.edu.pl; 2 Department of Civil Engineering, São Paulo State University (UNESP), Alameda Bahia, 550, Ilha Solteira 15385000, Brazil; v.figueiredo@unesp.br; 3 Civil Engineering Department, The University of Jordan, Amman 11942, Jordan; 4 Raw Building Materials and Processing Technology Research Institute, Housing and Building National Research Center (HBRC), 87 El-Tahreer St., Dokki, Giza, Cairo 12311, Egypt; hamdyabdelgawwad@yahoo.com; 5 Structural Engineering Department, Faculty of Engineering, Mansoura University, Elgomhouria St., Mansoura 35516, Egypt; mohamedattia@mans.edu.eg; 6 Building Materials and Construction Chemistry, Technische Universität Berlin, 13355 Berlin, Germany

**Keywords:** hot water curing, hot air curing, thermal curing, nanosilica, blast-furnace slag cement, strength, freeze-thaw, sorptivity, porosity, cement mortar

## Abstract

This investigation studies the effects of hot water and hot air curing on the strength development, transport properties, and freeze-thaw resistance of mortars incorporating low-heat blast furnace slag cement and nanosilica (NS). Mortar samples were prepared and stored in ambient conditions for 24 h. After demolding, mortar samples were subjected to two different hot curing methods: Hot water and hot air curing (40 °C and 60 °C) for 24 h. For comparison purposes, mortar reference mixes were prepared and cured in water and air at ambient conditions. Strength development (from 1 to 180 days), capillary water porosity, water sorptivity, and freeze-thaw resistance were tested after 180 days of curing. The experimental results showed that both curing regimes accelerate the strength development of mortars, especially in the first seven days of hydration. The highest early strengths were reported for mortars subjected to a temperature of 60 °C, followed by those cured at 40 °C. The hot water curing regime was found to be more suitable, as a result of more stable strength development. Similar findings were observed in regard to durability-related properties. It is worth noting that thermal curing can more efficiently increase strength in the presence of nanosilica, suggesting that NS is more effective in enhancing strength under thermal curing.

## 1. Introduction

Dynamic developments in industry at a worldwide level poses new challenges for the construction industry. The increase in the consumption of cement and concrete over the last decades, combined with increased environmental awareness, has necessitated the use of new types of cements [1]. Binary or ternary blended cement systems are increasingly being used to reduce carbon footprints [2]. One of the most popular of these, blast-furnace slag cement (CEM III), can be distinguished according to EN 197-1. As has been shown numerous times in the existing literature, CEM III has a positive effect in improving consistency and durability parameters. Moreover, thanks to its low hydration heat, it is more suitable in mass structures [3,4,5,6,7]. Elements made of slag cement are also more resistant to chemical aggression, such as that which is brought about by sulphates and chlorides [8]. Unfortunately, due to the presence of slag glass phases, such composites suffer from low early strength and hardening [8]. Despite the many advantages of concrete structures and their overall durability, exposure to harsh environments containing chloride ions, carbon dioxide, or sulphate can reduce their durability and performance over the years [9,10,11,12]. A generally accepted reason for why concrete is susceptible to chemical aggression is that its porosity and microstructural defects allow harmful ions to penetrate the surface into the inside of the concrete structure, ultimately resulting in cracks [13,14]. A common process of destruction involves sulphate penetration through the surface, which then reacts with hydrates and produces ettringite or gypsum, leading to volume expansion [15,16]. This creates tensile stresses in the concrete, which result in subsequent cracks. Thus, it is very important to produce composites with dense and compacted microstructures and satisfactory early mechanical performance.

Due to financial and technological reasons, high early strength is also demanded in the case of prefabrication technology. Acceleration of the hydration process, and thus strength gain, can be achieved by using the appropriate technical admixtures or special maturing techniques. There is no single fixed procedure and thus various approaches are presented in the literature, including steam curing, autoclaving, hot water curing, and hot air curing [17]. There is still no agreement as to the proper curing method and no consensus as to which one best guarantees satisfactory composite performance after curing. Some researchers suggest that the temperature curing process should take place after the initial set and that the temperature should not exceed 60–70 °C [18,19], while other researchers apply heating directly after casting [17]. 

The exposure of cementitious composites to elevated temperatures in the early ages causes internal water loss by self-desiccation and it is thus of key importance to facilitate water/moisture from external sources, during the heating process. Moreover, a thermally accelerated hydration process may result in non-uniform scattering of the hydration products, consequently creating very high porosity and cracking at later ages [17,18,20]. This phenomenon, often referred to as the crossover effect (COE), may lead to a decrease in the mechanical performance of composites at later ages [17]. To tackle this challenge, some authors have recommended that heating processes should not start directly after casting, i.e., within 24 h of curing [21,22]. Studies by Mardani et al. [23] and Wang et al. [24] have observed increases in compression strength during the first seven days, by using high temperature curing 24 h after casting. However, their results do not agree on subsequent strength. Sajedi and Razak [25] have reported that manipulating both the temperature and duration of heating enables effective control of early strength development, as well making it possible to avoid the crossover effect. 

Based on the available literature, it can be concluded that there is still no agreement on a proper thermal curing method. Moreover, most of the available studies are solely related to the strength development process, but this may be insufficient for establishing which curing technique is the most effective. As has been proven by Zou et al. [26], it is necessary to supplement tests of strength development processes with evaluations of durability-related properties, such as capillary water absorption. The capillary water absorption (CWA) of concrete reflects not only changes in the microstructure of the concrete surface, but is also indirectly related to its durability [27,28,29,30]. CWA correlates well with the internal pores of concrete, as well as its water sorptivity. Both of these parameters are often used to determine and compare the permeability of concrete [31,32,33]. Liu et al. [31] have indicated that CWA also correlates well with the durability indicators of concrete. 

Another approach for accelerating early strength development involves the incorporation of nanoparticles; i.e., calcium-silicate-hydrate seeds (C-S-H) or nanosilica (NS). It has been widely recognized that the use of nanosilica has a substantial effect on the acceleration of the cement hydration process, thus leading to faster setting and hardening of cementitious composites [34,35,36]. The beneficial effects of nanosilica on the properties of cementitious composites, in fresh and hardened states, can be attributed to three synergistic NS effects: Nucleating, filling, and high pozzolanic activity [37,38,39]. The effects of NS on the cement hydration process of Ordinary Portland Cement (OPC) and on binary cementitious systems under ambient [40,41] and elevated temperatures [42,43] have been confirmed by calorimetric studies. 

As far as the authors of this work are aware, research on the mechanical and durability performance of thermally cured binary cementitious systems, modified with silica nanoparticles, has been limited [44]. There is therefore a strong need to explore the effects of temperature curing on the long-term durability-related properties of cementitious composites. In addition, for precast concrete such technology is imperative in order to improve the efficiency and the characteristics of the precast elements for such aggressive conditions. This study aims to fill the gap in the existing knowledge and to shed light on the effects of temperature curing on the strength development, transport properties, and freeze-thaw resistance of low-heat blast-furnace slag cement mortars modified with nanosilica. Two different curing methods (hot air and hot water) and two heating temperatures (40 °C and 60 °C) were evaluated and compared with control specimens cured in ambient conditions (20 °C). 

## 2. Materials and Methods 

### 2.1. Materials

**Figure 1:**
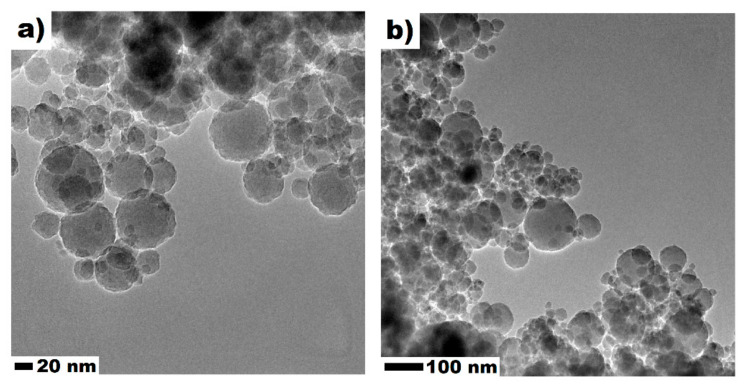

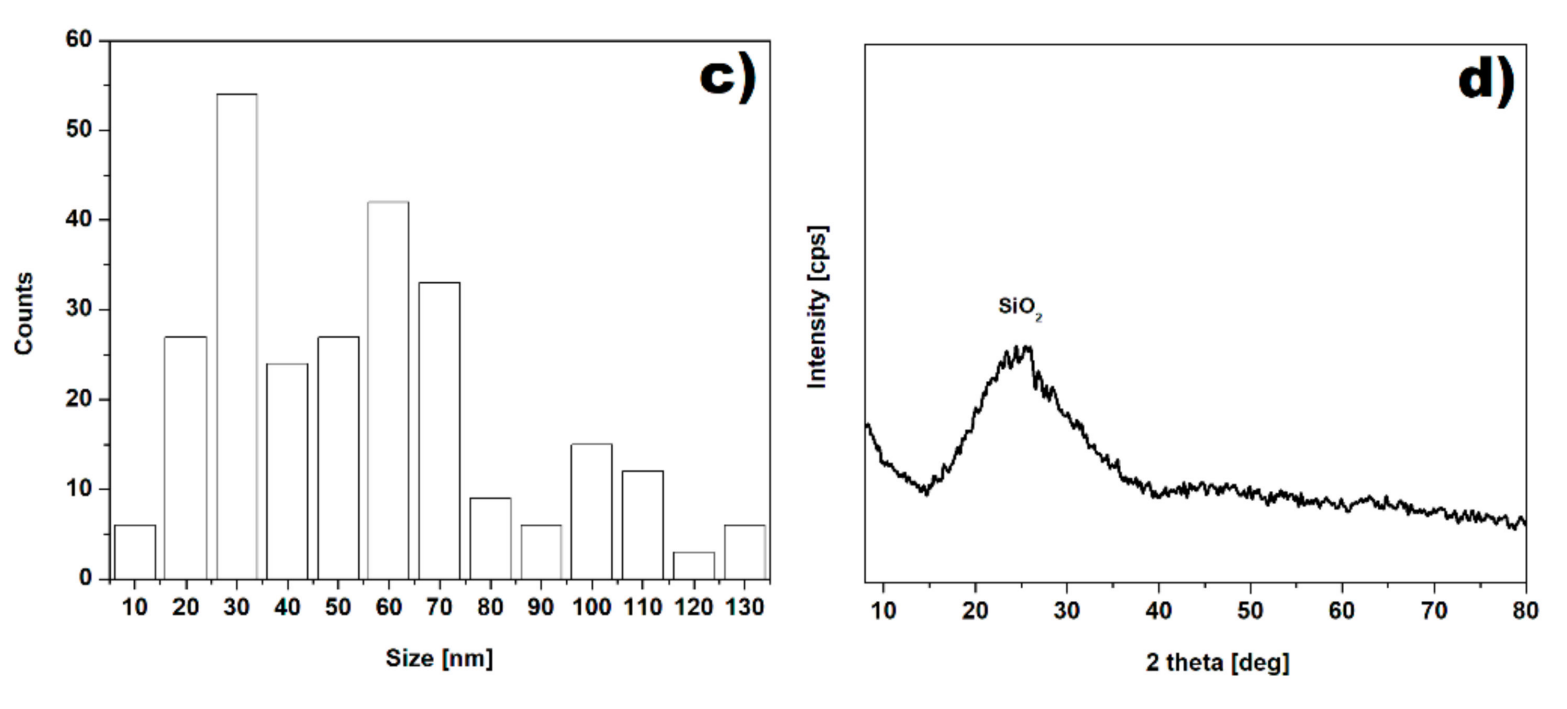
TEM images (**a**,**b**), size distribution (**c**), XRD (**d**) of nanosilica (NS). Reproduced from Sikora et al. 2020 [40].

**Figure 2:**
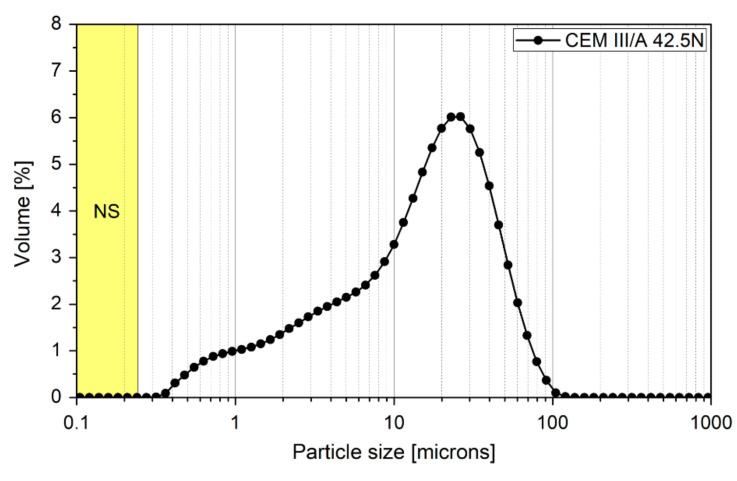
Particle size distribution of cement and river sand used in this study. The particle size range (based on TEM analysis) of silica nanoparticles has been highlighted in yellow.

In this investigation, low heat blast-furnace slag cement CEM III/A 42.5 N—LH/HSR/NA, produced by Górażdże (Chorula, Poland), river sand with particle sizes up to 2 mm (Bielinek, Poland), and tap water were used. Moreover, a silica colloidal suspension with a solid mass of 50 wt.-% was used. The general properties of the nanosilica (NS) are presented in Table 1. Figure 1a,b confirms the spherical shape of the silica nanoparticles, with X-ray diffraction confirming its high purity (Figure 1d). According to transmission electron microscopy (TEM) micrographs, NS particle sizes ranged 10–140 nm (Figure 1c). Detailed information regarding the nanosilica used in this study can be found in our previous work [40]. The particle size distribution (PSD) of the cement used in this study is presented in Figure 2. It can be clearly seen that NS was much finer than the particle size of the cement.

### 2.2. Mixture Design and Mixing Process

The mortar component proportions and the mixing procedure conformed to EN 196-1. The mortars were produced with a cement: River sand fixed ratio of 1:3. The w/c was fixed at 0.5. Reference mixtures (without nanosilica) were designated as series C, while mixtures containing nanosilica were designated as series N. Nanosilica suspension was mixed with water, prior to mixing with dry components. Based on our previous studies [40,41,45], 3% of nanosilica (by mass of cement) is the most effective dosage and it was therefore incorporated in the mortars. After mixing, the mortar samples were molded in 40 × 40 × 160 mm^3^ prisms for water accessible porosity and water sorptivity tests, while 40 × 40 × 40 mm^3^ cubical specimens were produced for compressive strength and freeze-thaw resistance tests. After 24 h of storing in ambient conditions, the specimens were demolded and subjected to various curing processes, which have been presented in the next section. In total, 72 prismatic and 576 cubic specimens were produced in this study. 

### 2.3. Experimental Protocol

**Figure 3:**
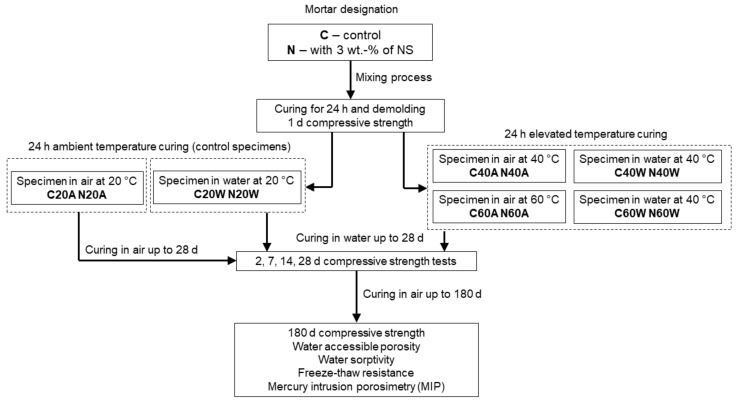
Experimental curing method protocol.

After 24 h of casting, the specimens were demolded and assigned to different curing conditions. In the research presented here, all samples were named according to a scheme in which the first letter designated whether it was a control (C) mix or a mix with nanosilica (N); the following number corresponded to the temperature at which a sample was cured, for between 24 h and 48 h (20/40/60); the last letter designated the type of curing (A—on air, W—in water). For instance, N40W refers to nanosilica-modified cement mortar cured for 24 h in water, at 40 °C. Series C40A, N40A, C60A, and N60A were wrapped in foil and exposed to air-oven curing for 24 h hours, at temperatures of 40 °C and 60 °C. Series C40W, N40W, C60W, and N60W were put in a sealed water tank and exposed to hot water curing for 24 h hours, at temperatures of 40 °C and 60 °C. After the heating procedures, the specimens were stored in water for up to 28 days of curing. Two series of mortars were prepared as control specimens. After demolding, mortars C20A and N20A were wrapped in foil and stored at a temperature of 20°C ± 2 °C and RH = 65 ± 5%, for up to 28 days. Mortars C20W and N20W were stored in a water tank at a temperature of 20°C ± 2 °C, for up to 28 days. After 28 days, the specimens were removed and cured for up to 180 days at ambient conditions (20°C ± 2 °C and RH = 65 ± 5%). A detailed experimental protocol is presented in Figure 3. 

### 2.4. Method

#### 2.4.1. Compressive Strength

The compressive strength of the mortars was determined with a digital crushing machine (Toni Technik -Zwick-Roell, Berlin, Germany), conforming to EN 196-1. Six cubes of each type of mortar mix were tested, with the mean value taken into consideration. After the planned curing period and at a specific age, the surfaces of the specimens were dried and the test was carried out.

#### 2.4.2. Capillary Water Porosity

The capillary water porosity of 40 × 40 × 160 mm^3^ mortar prisms was determined using the water displacement method, based on the Archimedes principle [46]. After 180 days of curing, the mass of saturated samples was measured under water (*m_sub_*). Afterwards, the surfaces of the specimens were dried and the mass of the saturated sample was determined (*m_sat_*). Finally, the specimens were dried at 105 °C to a constant mass and weighed (*m_dry_*), with the capillary water porosity calculated using the following formula (Equation (1)):

(1) $$Capillary\;water\;porosity\;\left[\%\right]=\frac{m_{sat}-m_{dry}}{m_{sat}-m_{sub}}\times 100$$

where, *m_sat_*, the mass of saturated sample, *m_dry_* is mass of the dry sample, and *m_sub_* is the underwater mass of the submerged sample.

#### 2.4.3. Water Sorptivity

**Figure 4:**
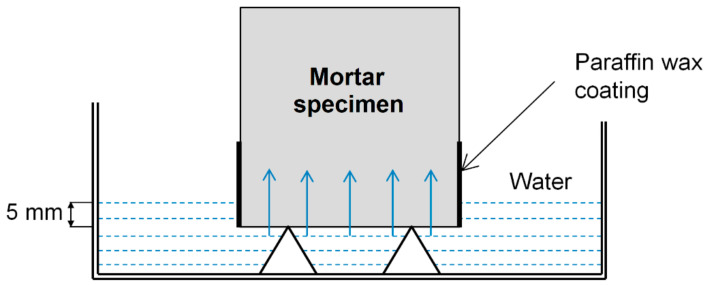
Schematic set-up for measuring the rate of water absorption in cement mortar specimens. Reproduced from [46].

The water absorption coefficient of the mortars was calculated based on the water sorptivity test, using the partial immersion method according to the guidelines of EN ISO 15148. Prior to the test, the sides of three 40 × 40 × 160 mm^3^ prisms of each type of mortar were sealed with hot paraffin wax. During measurement, the water level was kept constant at about 5 mm above the highest point of the bottom side of the prism (Figure 4). A detailed explanation of the procedure can be found in our previous work [46]. 

#### 2.4.4. Freezing and Thawing Resistance

**Figure 5:**
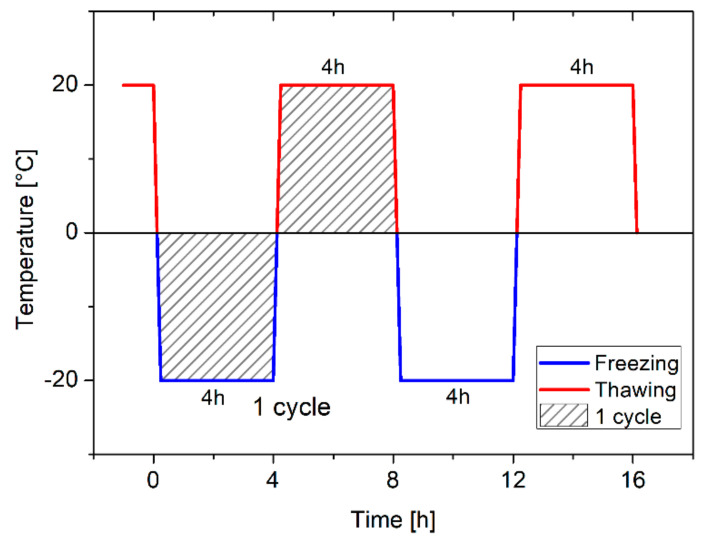
Freezing and thawing cycle.

Freeze-thaw resistance was determined according to the Polish Standard PN-85-B-04500 [47] (Figure 5). Firstly, 40 × 40 × 40 mm^3^ cubic specimens were used to determine the compressive strength of the mortars after the freezing and thawing test. After 180 days of curing, specimens were oven-dried at 105°C, until constant mass, and once again saturated in water for 7 days, after which they were exposed to 25 freeze-thaw cycles (group T25). The group T0 samples remained in water, at a temperature of 20 °C, without exposure to freezing and thawing. Basic freeze-thaw resistance was determined after 25 cycles, with each cycle lasting 8 h, during which the samples were frozen for 4 h in air at −20 °C and then thawed for another 4h in water at 20 °C. For each mix design, a total of 12 specimens were used; six specimens for group T0 and six for group T25 (25 cycles). In total, 144 specimens were tested with the freeze-thaw procedure. 

#### 2.4.5. Mercury Intrusion Porosimetry

Mercury intrusion porosimetry (MIP) was applied in order to assess the pore characteristics of the cement mortars. This is a common method widely used to characterize the pore structure of porous materials, including the cement-based and geopolymer composites used in construction [48,49]. Due to its simplicity, quickness, and wide pore diameter measuring range, it can be used as an indicator for understanding the durability related parameters of composites, including transport properties and freeze-thaw resistance [49]. MIP tests were performed on small-cored mortar samples taken out from the middle of the specimens. Prior to measurement, the samples were placed in a freeze dryer to stop hydration and remove pore moisture.

## 3. Results

### 3.1. Compressive Strength Development

**Figure 6:**
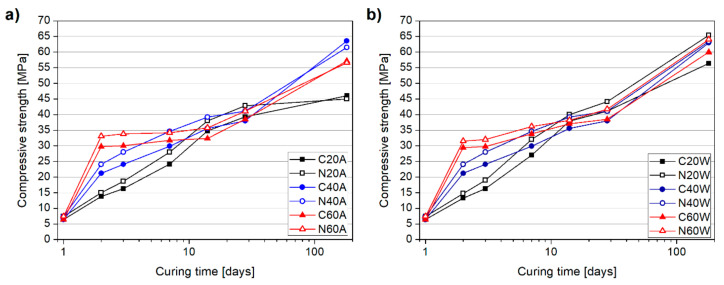
Specimens’ compressive strength development for up to 180 days of hot air curing (**a**) and hot water curing (**b**).

Figure 6 presents the experimental results of the compressive strength development of cement mortars at up to 180 days of curing. In general, a similar strength development trend can be seen with both techniques, for up to 28 days of curing, with curing temperature having a major influence. At an age of one day (after demolding), no differences between the compressive strength of the plain and NS-modified specimens were reported. However, from two days of curing on, a difference between the compressive strengths was observed, in favor of the N series. This effect can be attributed to the pozzolanic activity of nanosilica, which results in consumption of Ca(OH)_2_ and the production of additional C-S-H phase [40,50]. In regard to temperature, it can be clearly seen that the proposed curing methods have a significant effect on the early strength development of mortars. A temperature of 60 °C had the highest impact on early strength values, followed by a temperature of 40 °C. After temperature curing (two days), specimens C40A, N40A, C60A, and N60A exhibited 54%, 75%, 116%, and 139% higher strengths than the C20A specimen. Similarly, specimens C40W, N40W, C60W, and N60W exhibited 59%, 81%, 121%, and 137% higher compressive strengths than the C20W specimen. 

The incorporation of NS to the mixture was beneficial in the improvement of the compressive strength of specimens, with NS-modified specimens exhibiting higher compressive strengths than the corresponding control mortars; the effect was more pronounced in specimens that were thermally cured. The increase in the strength with nanosilica addition is in the range of 5–20% depending on the mortar age as well as the applied curing regime. This observation is in line with Mei et al.’s [44] study, where it was found that a combination of nanosilica and temperature curing promotes cement hydration. In general, the discrepancy between the compressive strength values attributed to thermal curing are visible for up to 14 days of curing. Afterwards, considerable differences between specimens were not observed. After 28 days of curing, specimens containing NS exhibited only a slight strength improvement; on average by 8%. A review of the literature shows that NS exhibits noticeable effects, both on early and later strength development, although most studies have focused on OPC systems. However, in our case, low heat blast-furnace slag cement was used and thus the reaction rate of NS and cement was most probably slightly lower than in a pure OPC system. Moreover, irrespective of the curing method used, mortars exhibited only a minimal strength decrement, not exceeding 8%. It was thus confirmed that, when thermal curing is applied after 24 h of hydration, rather than directly after casting, the crossover effect is limited. 

**Figure 7:**
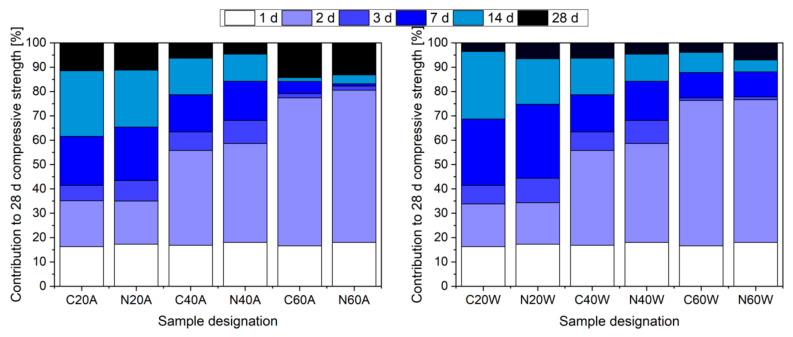
Development of strength of hot air-cured (**left**) and hot water-cured specimens (**right**).

Figure 7 presents strength development in days (age). It can be clearly seen that the heating process (2 days) made a major contribution to the 28 days compressive strength. The effect was more pronounced with an increment in temperature and it can be seen that hot air curing made a higher contribution to 28 days strength, than the water curing method. Nevertheless, despite noticeably higher acceleration of strength development, compressive strength development in hot air cured specimens in later ages was impeded, in favor of the hot water cured specimens. This was especially visible at 14 days, where all hot water cured specimens almost reached the 28 days strength, while specimens C60A and N60A reached less than 90% of 28 days of compressive strength. Incorporation of nanosilica in the mixture was found to have a beneficial effect on accelerating the strength gain of specimens in ambient conditions, as well as during exposure to a temperature of 40 °C.

To determine the possibility of the presence of the crossover effect, the compressive strengths of mortar specimens were additionally measured after 180 days of curing (Figure 6). After 180 days of curing, the choice of curing method seems to have a much more pronounced effect on the compressive strength, than at 28 days. This can be attributed to fact that low heat blast-furnace slag cement was used, which is widely known to have a relatively slow hydration process. In the case of the hot air curing method, it can be seen that specimens C20A and N20A exhibited the lowest strengths in this group. This can be attributed to the fact that, after demolding, specimens were cured in air conditions and thus a limited amount of moisture was available for hydration. On the contrary, specimens C40A, N40A, C60A, and N60A, after air curing process, were stored underwater until 28 days of age (Figure 3). In the case of hot air cured specimens, the best performance was achieved by specimens exposed to a temperature of 40 °C (C40A and N40A), followed by those exposed to a temperature of 60 °C. Despite the noticeable effects of nanosilica in the early days of hydration, its effect on the compressive strength of the specimens after 180 days was negligible. 

In the case of the water-cured specimens, all mortars besides C60W and C20W exhibited comparable strengths, with the highest value reported for N20W. The compressive strength values of N20W, C40W, N40W, and N60W were comparable to those of specimens that exhibited the best performance under hot air curing; namely, C40A and N40A. Similar to the case of the air curing method, mortars with and without NS exposed to hot water curing exhibited comparable strength values. In contrast, the control specimens, C20W and N20W, exhibited a discrepancy in their results, in favor of the NS-modified specimen, which exhibited 16% higher strength. 

Based on the compressive strength results, it can be concluded that the hot water curing method facilitates more uniform strength development than oven-air curing. In addition, both temperatures of 40 °C and 60 °C seem to be suitable for enhancing the early strength development of low-heat cement mortars. Furthermore, in both curing procedures, nanosilica is beneficial in accelerating strength development, but at 28 days and 180 days, the beneficial effect of nanosilica is negligible. 

### 3.2. Transport Properties

**Figure 8:**
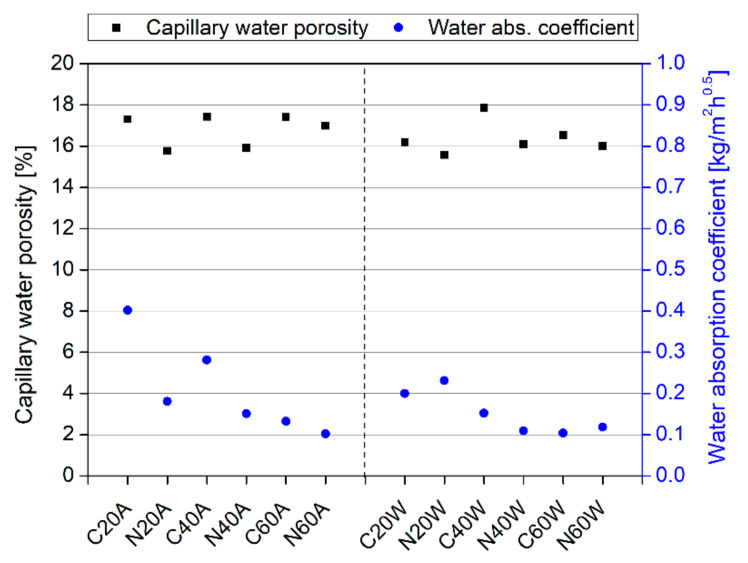
Transport properties of cement mortars after 180 days of curing.

Figure 8 presents the results of tests of capillary water porosity and the water absorption coefficient of mortar specimens after 180 days of curing. In general, it can be seen that thermal curing had beneficial effects on reducing the mortars’ transport properties. However, the hot air cured specimens exhibited slightly worse transport properties than the water cured ones. In addition, significant improvements were found for specimens containing nanosilica. Both the results of capillary water porosity and water absorption showed a similar trend. After exposure to a temperature of 60 °C, the difference between the NS-modified and control specimens was limited, confirming that a major influence on modification properties is temperature exposure. Higher discrepancies can be discerned for air-cured specimens, while lower ones were seen in the case of hot water cured specimens. The absorption coefficients of water cured samples were lower than that of air cured ones. This influence was less pronounced for capillary water porosity. It can also be clearly seen that the water absorption coefficient decreased with capillary porosity. Additionally, the water absorption coefficient decreased with increasing curing temperature, in both the hot air and hot water cases. For both properties it is clear that incorporation of nanosilica has an influence in reducing the capillary porosity as well as the water absorption coefficient in both cases: Air curing and water curing. In most mixes, direct relation between capillary porosity and transport properties can be caught. However, for some mixes N20W and C40W, the absorption coefficient is increasing with reducing the capillary porosity in the first and vice versa in the second mix. This might be attributed to the differences in the pore structures, which cannot be measured by the water sorptivity test. 

### 3.3. Freezing and Thawing Resistance

**Figure 9:**
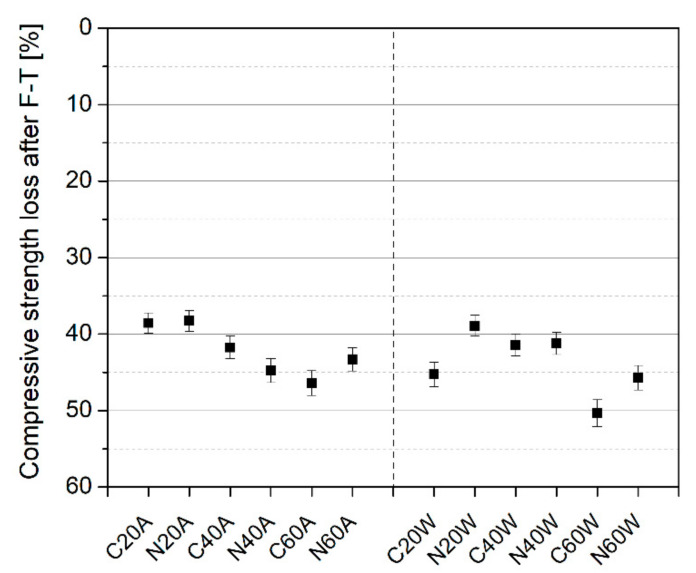
Compressive strength of mortar specimens, after exposure to freeze–thaw cycles.

The influence of freezing and thawing on the compressive strength of mortars, after 180 days of curing, is presented in Figure 9. It is clear that, for the first series with hot air curing, as the curing temperature increased, the strength deterioration after exposure to freezing and thawing increased gradually. The control mix for the first series (C20A) lost about 40% of its original strength, due to exposure to freezing and thawing cycles. However, by increasing the curing temperature to 60 °C, strength loss increased to about 48% (C60A). The inclusion of NS had a marginal influence on strength loss after exposure to freezing and thawing in air-cured specimens. In contrast, a different trend was observed in water-cured specimens. The incorporation of nanosilica appeared to improve the resistance of mortars, subjected to water curing, to freezing, and thawing. This observation is in line with the available literature [51,52]. A large difference between C40W and N40W was not reported, but these specimens exhibited substantially improved freezing and thawing resistance, as compared to the C20W specimen. On the other hand, increasing the curing temperature to 60 °C resulted in a slight decrement in residual strength, after exposure to freezing and thawing (C60W), although this was partially suppressed by the presence of NS (N60W). It can also be seen that, in both groups, as the curing temperature increased, the residual strength, after the freezing and thawing cycles, was significantly reduced. This can be attributed to the influence of high temperature curing in accelerating the hydration of the cement component randomly. The hydration products thus formed are weaker than the ones formed without heat curing. However, the hydration degree of cement is higher, which is reflected in compressive strength results before the test. When the samples were exposed to freezing and thawing cycles, fast deterioration occurred in all of them, but it was more pronounced in samples cured at high temperatures, due to the weakness of the C-S-H phases formed. The role of nanosilica in reducing strength deterioration, after exposure to freezing and thawing, is more detectable in the group of hot water cured specimens, as can be seen in Figure 9.

### 3.4. Mercury Intrusion Porosimetry (MIP)

**Figure 10:**
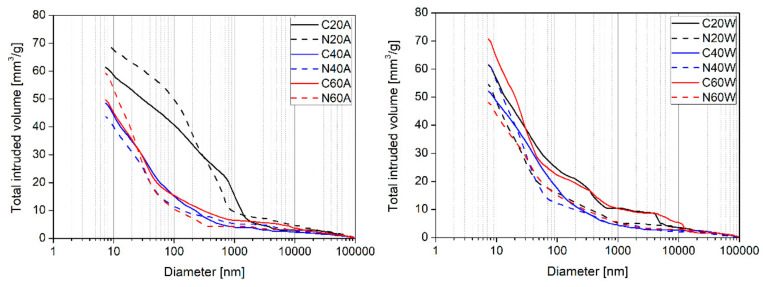
Cumulative intruded pore volume versus pore diameter of hot air-cured (**left**) and hot water-cured specimens (**right**).

Figure 10 and Table 2 present the results of MIP tests, after 180 days of curing. In general, water curing was more efficient than air curing in all the mixes. In addition, the incorporation of nanosilica reduced the porosity of most mixes, compared to the reference mixes. Regarding the curing methods, it is clear that for most mixes, the total porosity decreased with increasing heating temperature. For example, the porosity of C20A decreased from 13.05% to 10.35%, when cured with hot air at a temperature of 40 °C (C40A). Similarly, for the hot water curing group, porosity decreased from 12.24% (C20W) to 10.67% at 40 °C; the only exception being mix C60W, where the porosity increased with increasing curing temperature. However, noticeable differences in the average and median pore diameters, between specimens, can be distinguished (Table 2). Specimens C20A and N20A exhibited significantly higher porosities, due to the fact that they were directly air-cured after demolding; limited moisture was thus available during the hydration process. Important differences in the curve characteristics can be distinguished (Figure 10): The curves of the NS-modified specimens shifted noticeably to a region of smaller pores, which confirms that the incorporation of NS has substantial effects on pore structure alteration. This was reflected in the decrement of the average and median pore values in the NS-modified specimens (Table 2). This study therefore confirms the observations of other researchers [53], that the incorporation of NS in a mixture does not necessarily have to have an impact on the decrement in cement matrix porosity, but that it has substantial effects on pore structure alteration. As has been reported by other researchers, the presence of nanosilica in cementitious composites results in the disconnection of continuous pores from discontinuous ones, the subdivision of larger pores into smaller ones, as well as in the modification of the their shapes and tortuosity [54,55]. This could explain the substantial improvement of transport properties in mortars containing nanosilica (Figure 8). Nevertheless, the correlation between the results of MIP and freezing and thawing resistance cannot be established directly.

## 4. Conclusions

The following conclusions can be drawn from this work:

- The proposed curing methods have a considerable effect on the early strength development of low heat blast-furnace slag cement mortars. A strength development trend, comparable to 28-day curing, was noticed when both curing techniques were applied, with curing temperature having a major influence. A temperature of 60 °C had the highest impact on the early strength values, followed by a temperature of 40 °C.
- Based on the compressive strength results, it can be concluded that the hot water curing method facilitates more uniform strength development than oven-air curing. In addition, both temperatures (40 °C and 60 °C) were found to be suitable for enhancing the early strength development of low heat cement mortars. Moreover, irrespective on the curing method, mortars exhibited minimal strength decrement. Thus, it was confirmed that, when thermal curing is applied after 24 h of hydration and not directly after casting, the crossover effect is limited.
- The incorporation of nanosilica in mixtures was found to have a beneficial effect in accelerating specimens’ strength gain in ambient conditions, as well as during exposure to a temperature of 40 °C. However, at a temperature of 60 °C, the beneficial effects were limited, with the major contribution being temperature. NS-modified specimens exhibited higher strength gains, after thermal curing, than corresponding control (pristine) specimens.
- Due to a substantially accelerated cement hydration process and thus early strength gain associated with the synergistic effects of nanosilica and temperature, the effect of nanosilica on compressive strength at 28 days and 180 days was found to be negligible.
- Thermal curing had beneficial effects on improving the transport properties of mortars. The hot air cured specimens exhibited slightly worse transport properties than hot water cured specimens. In addition, significant improvements were reported in specimens containing nanosilica. MIP studies confirmed that NS has substantial effects on refinement of the pore structure in a cement matrix.
- A slight decrement in the freezing and thawing resistance of thermally cured specimens was found, but these changes did not exceed 10%. The incorporation of NS improved the resistance of mortars subjected to water curing, to freezing, and thawing.

## Figures and Tables

**Table 1:** Properties of colloidal silica.

Particle Size	Solid Content	Density	Viscosity	pH
10–140 nm *	50 wt.-%	1.4 g/cm^3^	8 cP	9.5

* based on TEM analysis.

**Table 2:** Porosities, average pore diameter, and median diameter of the cement mortars after 180 days of curing.

Mix	C20A	N20A	C40A	N40A	C60A	N60A	C20W	N20W	C40W	N40W	C60W	N60W
Total intruded vol. [mm^3^/g]	61.60	70.83	48.77	43.94	50.21	59.56	61.75	55.06	52.47	62.25	71.09	48.69
Porosity by Hg intrusion [%]	13.05	13.78	10.35	9.38	10.03	12.22	12.24	11.12	10.67	12.75	13.46	9.86
Average pore diameter [nm]	51.61	62.52	26.28	25.46	25.94	25.82	28.61	22.58	29.96	27.8	25.45	26.41
Median pore diameter [nm]	256.12	216.3	40.46	34.66	36.47	25.1	49.84	28.46	49.01	52.96	32.8	38.53
